# A case report: Treating insomnia with olanzapine in cancer patients

**DOI:** 10.1017/S1478951525100345

**Published:** 2025-06-20

**Authors:** Zhu Wang, Kimberson Tanco, Eric Liu, Sahithi Madireddy, Kathleen Renee Warner, Thomas J. Smith

**Affiliations:** 1 Section of Palliative Medicine, Division of General Internal Medicine, Johns Hopkins Hospital, Baltimore, MD, USA; 2 Department of Palliative Care and Rehabilitation Medicine, Division of Cancer Medicine, The University of Texas MD Anderson Cancer Center, Houston, TX, USA; 3 The Johns Hopkins University School of Medicine, Baltimore, MD, USA

**Keywords:** Insomnia, olanzapine, cancer, case report, palliative medicine

## Abstract

**Objectives:**

Patients with cancer frequently experience insomnia that significantly impacts their quality of life, worsens existing symptoms, and potentially hinders treatment outcomes and recovery. Here, we report on 3 cancer patients whose insomnia was improved with low-dose olanzapine.

**Methods:**

A retrospective review of medical records was conducted for 3 cancer patients experiencing insomnia treated with olanzapine at Johns Hopkins Hospital. The data collection included the type of cancer diagnosis, the level of insomnia severity experienced by individuals, and treatment results and outcome.

**Results:**

Olanzapine improved sleep in all 3 patients and decreased nausea/vomiting and anxiety in patients 2 and 3.

**Significance of results:**

A low dose of olanzapine has potential to treat insomnia in cancer patients. The ideal dosing regimens and potential risks are unclear, especially for long-term use. More research and clinical trials are needed to evaluate off-label use of olanzapine for insomnia, including its efficacy and risks, and to optimize the dosage to reduce its side effects in cancer patients. Oncology providers should consider olanzapine as a potential treatment for insomnia, especially given its off-label uses and potential benefits.

## Introduction

Patients with cancer experience a variety of physical, emotional, and psychological symptoms (Esther Kim et al. 2009). A common and highly distressing symptom is insomnia, which is characterized by difficulty falling asleep and/or staying asleep. It is 3 times more common in cancer patients than in the general population, and as many as 50% of cancer patients experience insomnia (O’Donnell 2004). Sleep is extremely important, especially for patients fighting cancer, as it allows their body to repair and rebuild, supports their immune system, helps manage pain and fatigue, and is crucial for maintaining mental well-being. Lack of sleep can significantly affect quality of life and negatively affect their ability to cope with cancer and its side effects (Palesh et al. 2010). However, insomnia has received minimal attention when compared with other symptoms such as pain and fatigue (O’Donnell 2004).

Olanzapine, an antipsychotic, is often used off-label to treat anxiety, anorexia, nausea, vomiting, and insomnia in cancer patients (Dev et al. 2023; Mukhopadhyay et al. 2021). When compared with other antipsychotics, olanzapine has lower rates of discontinuation and extrapyramidal symptoms and average amounts of QTc prolongation, but it has the highest level of sedative effects (Leucht et al. 2013). The sedative effects of olanzapine are thought to be due to its ability to block dopamine, serotonin, and histamine receptors (Bymaster et al. 1996; Zhang and Bymaster 1999). By blocking these receptors, olanzapine can decrease the activity of these neurotransmitters, leading to a calming and sedating effect. Furthermore, olanzapine has a much longer half-life than medications commonly used for insomnia, such as benzodiazepines, allowing individuals to sleep throughout the night (Miller 2004). In addition to the sedative effects, olanzapine can increase the duration of rapid eye movement sleep, which is often disrupted with insomnia (Gimenez et al. 2007). It also improved both sleep initiation and maintenance. However, unlike benzodiazepines, it does not potentiate respiratory depression when used in conjunction with opioids (Davis and Sanger 2020), which is utilized to manage pain in cancer patients.

Here, we present 3 cases of patients with cancer who experienced insomnia and were treated successfully with olanzapine. The outcomes suggest that olanzapine has the potential to be used for insomnia management in cancer patients.

## Case presentation

### Patient 1

A female in her 60s presented with recurrent abdominal pain and was found to have a mass causing intestinal obstruction. She was subsequently diagnosed with metastatic gastric adenocarcinoma and was trialed on 5FU/LV (FOLFOX) and oxaliplatin, and gastric stents were placed to relieve her obstruction. Palliative care was requested for goals of care discussion and symptomatic management. For symptoms of nausea, vomiting, anxiety, and insomnia, she was prescribed olanzapine 5 mg at bedtime as she had developed tolerance to granisetron patch, ondansetron, and metoclopramide. She was trialed on a course of zolpidem but it was weaned off for unknown reasons. With Edmonton Symptom Assessment System evaluation, her anxiety and nausea scores did not change at 5/10; however, her insomnia score decreased from 6/10 to 0/10 after 4 days of olanzapine treatment and remained at 0 until death. Her BMI was 25.58 at the initiation of olanzapine and increased to 31.62 a few months later. She was enrolled in home hospice and was comfortable at home with family. The etiology of the increase in BMI is unclear.

### Patient 2

A man in his 60s with metastatic prostate cancer status post proctectomy and adjuvant hormonal therapy presented with uncontrolled abdominal pain, anxiety, poor appetite, and insomnia. He received bicalutamide and leuprorelin and enzalutamide with adjuvant radiation therapy. Follow-up PET-CT scan showed the progression of disease. He rated his insomnia as moderate (2/3). After 12 days with olanzapine 5 mg at bedtime and increase of his opioid medication, his anxiety and insomnia were improved to mild (1/3). After successful control of his symptoms, he was looking forward to return to home. His BMI at the time of olanzapine initiation was 26.5 and 31.32 after a couple of months. The increase in BMI may be related to hormonal therapy and olanzapine.

### Patient 3

A female in her 40s had a diagnosis of inferior vena cava leiomyosarcoma underwent a resection of her tumor with a right-sided nephrectomy. She has been unsuccessfully trialed on the several lines of chemotherapy and radiation. Her chemotherapy resulted in nausea and vomiting. Treatment with prochlorperazine, ondansetron, and dexamethasone was all ineffective. In addition, she had anxiety that was unable to be controlled by alprazolam. For the symptoms described above, as well as poor appetite and moderate insomnia, she was started on olanzapine 5 mg daily. Olanzapine quickly improved her nausea and vomiting, appetite, and anxiety. After a few months with olanzapine, her insomnia has improved significantly, transitioning from a moderate degree (2/3) to a level of 0, indicating complete resolution of sleep disturbances. Her BMI was 20 at the time of olanzapine initiation but continued to downtrend until her time of death with a final BMI of 16.58.

## Discussion

Regardless of cancer type, patients often experience insomnia. Addressing insomnia in cancer patients is not only about improving sleep but also about improving their overall health, well-being, and ability to cope with the challenges of cancer treatment and survivorship. Olanzapine is increasingly used off-label for insomnia in cancer patients due to its sedative effects (Dev et al. 2023; Felton et al. 2016). Olanzapine’s sedative effects are primarily attributed to its antagonism of dopamine, serotonin, and histamine receptors (Miller 2004; Navari 2014) and thus often used off-label in palliative medicine for cancer-related insomnia. There is limited research evaluating the effects of olanzapine on insomnia. One previous study showed that olanzapine has long-term efficacy in managing chronic insomnia (Khaledi-Paveh et al. 2021).

Patient 1 presented with severe nausea/vomiting, anxiety, and insomnia. She was prescribed zolpidem, a non-benzodiazepine, for treating insomnia; however, it was weaned off for reasons not stated in her chart. Olanzapine at 5 mg resulted in complete improvement for insomnia in 4 days despite having no effects for nausea/vomiting and anxiety. This case suggests that olanzapine at low dose is effective in treating insomnia in patients with cancer. Other non-benzodiazepine medications for insomnia, such as zolpidem, should be avoided in elderly as they can significantly increase the risks of fall and result in severe injuries.

Patient 2 used opioids to treat severe pain. Olanzapine was a good option to treat his insomnia as it can improve sleep quality and potentially reduce opioid withdrawal symptoms (2014; Davis and Sanger 2020; Khaledi-Paveh et al. 2021). In addition, olanzapine is not considered a significant respiratory depressant when combined with opioids unlike benzodiazepines (Boon et al. 2020). Therefore, olanzapine would be a better choice for insomnia in cancer patients who are concurrently using opioids.

Patient 3 presented with severe nausea/vomiting, anxiety, and insomnia. A standard combination of antiemetic drugs and an anti-anxiety drug failed to control her symptoms. Olanzapine at 5 mg/day substantially improved all of her symptoms in a short period of time. After a few months, her insomnia improved from moderate to 0, and her appetite also improved.

To our knowledge, there are no formal studies to evaluate the use of olanzapine for insomnia in cancer patients. A small double-blind, randomized, placebo-controlled trial has shown that olanzapine can improve sleep efficiency, including total sleep time and sleep latency, in patients with depression and bipolar disorder (Lazowski et al. 2014). However, adverse effects such as drowsiness, hypotension, neutropenia, and increased restlessness have been reported (Dev et al. 2023). Although benzodiazepines, non-benzodiazepines (like zolpidem), and olanzapine are all used to treat insomnia, they carry different benefits and risks (Lie et al. 2015). Benzodiazepines can be effective for both sleep onset and maintenance, but they are generally discouraged in cancer patients due to increased risk of respiratory depression with concurrent use of opioid (Guina and Merrill 2018). Other risks of using benzodiazepine include dependence and risk of misuse, and withdrawal, making it a poor choice to use long-term (Soyka et al. 2023). Non-benzodiazepines, like zolpidem, have the same risks as benzodiazepines and is associated with high risks of falls in elderly patients (Wagner and Wagner 2000). Olanzapine, an antipsychotic, is often used off-label for insomnia and can be effective for both sleep initiation and maintenance. Although olanzapine has the risks of significant metabolic disturbances and weight gain (Cederlof et al. 2024), it can be minimized and sometimes eliminated by reducing its dosage and duration of usage. The weight gain with olanzapine appears to be a potential option for improving appetite and weight gain in cancer patients who have low BMI. The treatment duration needs to be determined based on several factors, including the specific condition being treated, the individual’s response to treatment, and the type of intervention being used. The use of medications is both a science and an art, which needs to be adjusted for each individual patient to maximize benefits and minimize risks.

## Conclusion

Olanzapine’s sedative effects can be beneficial for patients experiencing insomnia. In all 3 cases reported, olanzapine improved insomnia even at a low dose. In addition, it improved other common symptoms, including nausea, anorexia, and anxiety. No formal study has assessed its efficacy and risks in cancer patients, especially with long-term use. More research is needed to evaluate the balance between potential benefits and risks of olanzapine toward a goal of treating insomnia with a safe dosage in patients with cancer.
